# Association between KIF6 rs20455 polymorphism and the risk of coronary heart disease (CHD): a pooled analysis of 50 individual studies including 40,059 cases and 64,032 controls

**DOI:** 10.1186/s12944-017-0651-y

**Published:** 2018-01-05

**Authors:** Yan Li, Zhen Chen, Hejian Song

**Affiliations:** 1 Heart Function Examination Room, the First People’s Hospital of Lianyungang, Affiliated Hospital of Xuzhou Medical University, Lianyungang, Jiangsu 222002 China; 2 Department of Neurosurgery, the first People’s Hospital of Lianyungang, Lianyungang, Jiangsu 222002 China; 3 Department of Cardiology, the First People’s Hospital of Lianyungang, Lianyungang, Jiangsu 222002 China

**Keywords:** Coronary heart disease, KIF6 rs20455, Polymorphism, Meta-analysis

## Abstract

**Background:**

The KIF6 rs20455 polymorphism has been verified as an important genetic factor of coronary heart disease (CHD), but with controversial results. The aim of this study was to explore the association between KIF6 rs20455 polymorphism and susceptibility to CHD.

**Methods:**

All eligible studies were identified by searching Medline (mainly PubMed), EMBASE, the Web of Science, Cochrane Collaboration Database, Chinese National Knowledge Infrastructure, Wanfang Database and China Biological Medicine up to October 5, 2016.Odds ratios (ORs) with 95% confidence interval (CI) were used to explore the association between KIF6 rs20455 polymorphism and CHD risk. Begg’s and Egger’s tests were used to examine the publication bias. Subgroup analysis and sensitivity analysis were performed to test the reliability and stability of the results. All the analyses were carried out by Stata 12.0 software.

**Results:**

A total of 28 publications including 50 individual studies were analyzed in this present work. There are no significant association found between KIF6 rs20455 polymorphism and CHD risk (Homozygote model: OR = 1.007, 95% CI =0.952–1.066, *P* = 0.801; Heterozygote model: OR = 1.009, 95% CI = 0.968–1.052, *P* = 0.636; Dominant model: OR = 1.007, 95% CI = 0.966–1.048, *P* = 0.753; Recessive model: OR = 0.989, 95% CI = 0.943–1.037, *P* = 0.655; Allele comparison model: OR = 1.00, 95% CI = 0.971–1.030, *P* = 0.988). Furthermore, subgroup analyses were performed by ethnicity, source of control.

**Conclusions:**

Our result suggests that KIF6 rs20455 polymorphism may not be associated with CHD susceptibility. However, additional very well-designed large-scale studies are warranted to confirm our results.

## Background

Coronary heart disease (CHD), a multifactorial heart disorder resulting from both environmental and genetic factors [1], is one of the leading causes of disability and death around the world [2]. Epidemiology studies have suggested that hypertension, hyperlipidemia, diabetes mellitus, obesity and smoking are major risk factors for CHD [3]. In recent years, more and more studies reveled that several loci and variants are strongly associated with CHD [4, 5]. It has been estimated that approximately 50% of the variability of the major risk factors for CHD is determined by genetics [6].

The KIF6 protein is one of several molecular components that mediate intracellular transport of organelles, protein complexes, and mRNAs. A common Trp719Arg (rs20455) SNP in exon 19 of the KIF6 gene has been identified as a potential risk factor for CHD [7, 8]. The KIF6 protein belongs to the kinesin superfamily, which is involved in the intracellular transport in a microtubule and ATP-dependent manner [9]. The rs20455 polymorphism replaces the nonpolar ‘Trp’ residue in codon 719 with a basic ‘Arg’ amino acid. This SNP lies near the putative cargo binding taildomain and may alter the cargo activity of KIF6 [10]. Carriers of the 719Arg allele exhibit a 50% increased risk of events compared with non-carriers [8, 11]. Up to now, multiple large prospective and case–control studies have reported the association between KIF6 rs20455 polymorphism and the risk of CHD. However, somestudies have not verified inconsistent results. Published studies have generally been restricted in terms of sample size and ethnic diversity, and individual studies may have in-sufficient power to achieve a comprehensive and reliable conclusion. In view of the discrepancies in the findings of previous published studies, we aimed to perform a meta-analysis of the published studies to clarify the association between KIF6 rs20455 polymorphism and CHD to get a better under-standing of this relationship.

## Methods

### Literature search

A comprehensive search for all related studies from both electric databases, such as, Medline (mainly PubMed), Embase, Web of science, China National Knowledge Infrastructure (CNKI) et al., and hand search from references of all eligible literatures. Single or combinations of the following keywords were used: “kinesin like protein 6” or “KIF6” or “rs20455” or “719Arg”, “single nucleotide polymorphism, SNP or variation, mutation”, “genetic association” and “coronary heart disease” or “CHD”. No language and sample size were set. When more than one studies of the same population were included in several publications, only the most recent or complete studies were included in this meta-analysis.

### Selection criteria

Articles included should meet following criteria: an appropriate description of KIF6 rs20455 polymorphism in CHD cases and healthy controls; results expressed as odds ratio (OR); and studies with a 95% confidence interval (CI) for OR with sufficient data to calculate these numbers. While for the exclusion criteria provided as follows: studies without raw data; case-only studies, family-based studies, case reports, editorials, and review articles (including meta-analyses). In studies with overlapping cases/controls, the study with the higher quality score or the study with more information on the origin of the cases/controls was included in the meta-analysis.

### Data extraction

Two researchers extracted important information independently and carefully from all eligible studies according to the criteria listed above. Any disagreement will be resolved by the two authors through discussion or the third author. The following data were extracted from each included study: first author’s surname, year of publication, country, ethnicity, genotyping method, source of control, total number of cases and controls, distributions of KIF6 rs20455 genotypes. Different ethnicity descents were categorized as Caucasian, Asian, and Mixed populations (the original studies didn’t clarify the race of the subjects or mixed races).

### Statistical analysis

We adopted poled ORs and corresponding 95% confidence interval (CIs) to detect the association between KIF6 rs20455 polymorphism and CHD risk. Heterogeneity was explored by Q statistic [12], and the *P* value was <0.05 will be considered statistically significant. Heterogeneity was also assessed using the *I*^*2*^ statistic, which takes values between 0% and 100% with higher values denoting greater degree of heterogeneity (*I*^*2*^ = 0–25%: noheterogeneity; *I*^*2*^ = 25–50%: moderate heterogeneity; *I*^*2*^ = 50–75%: large heterogeneity; *I*^*2*^ = 75–100%: extreme heterogeneity) [13]. Different statistical models will be selected according to the result of heterogeneity. Random (Der Simonian-Laird method) [14] will be used to calculate the precise results when the *P* value of heterogeneity was <0.05, or the *I*^*2*^ > 50%. Otherwise, fixed effects model (Mantel-Haenszel method) will be adopted [15]. Five genetic comparison model were carried out and calculated as follows: homozygote model (GG vs. AA), heterozygote model (AG vs. AA), recessive model (GG vs. AG + AA), and dominant model (GG + AG vs. AA), and allele comparison model (G-allele vs. A-allele). Hardy–Weinberg equilibrium in the control group was tested by the chi-square test for goodness of fit, and a *P* value of <0.05 was considered significant. Subgroup analyses were performed by ethnicity, source of control, to confirm if our results were stable and robust [16]. Begg’s funnel plots [17] and Egger’s test [18] were explored to examine if potential publication bias were existed in this study. Sensitivity analysis was carried out by sequentially omitting each study and finding the influence on the overall summary estimate [19]. All the statistical analyses were finished by STATA software (version 12.0; Stata Corporation, College Station, TX). All the *P* values were two-sided.

## Results

### Characteristics of all included studies

**Fig. 1:**
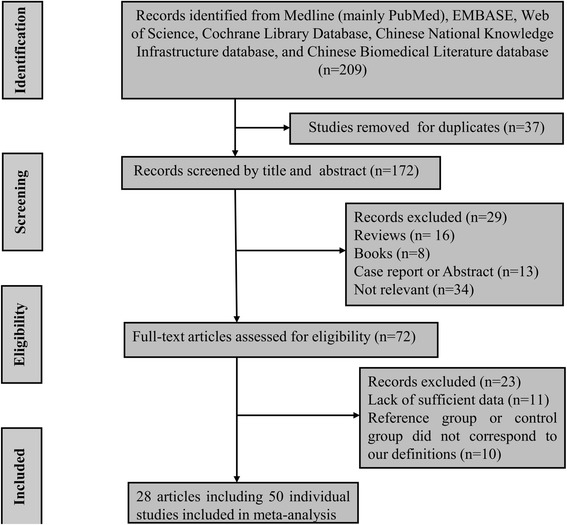
The process of literature research

Totally, 209 potential relevant studies were searched through several databases. Based on the including criteria listed above, only 28 articles including 50 separate studies were included finally [8, 20–46]. A flow diagram summarizing the process of study selection was present in Fig. 1. The baseline characteristics ofall included studies were listed in Table 1. Helgadottir et al. contained two individual studies [25], Samani et al. contained two individual studies [26], Assimes et al. contained 20 individual studies [31], and Wu et al. contained two separate studies [41]. Moreover, there were 37 studies from Caucasian decedent, 9 studies from Asian populations and the rest 14 studies from mixed populations. There were 20 population-based (PB) studies, 21 hospital-based (HB) studies and four family based (FB) study, three community based (CB) study, two hospital and community based (H-CB) study. Different ethnicity descents were categorized as Caucasian, Asian and Mix (the original studies didn’t clarify the race of the subjects or mixed races).

**Table 1:** Characteristics of all studies included in this meta-analysis

Author	Year	Country	Ethnicity	Control source	Case	Control	Case			Control			*P*_*HWE*_
							AA	AG	GG	AA	AG	GG	
Berglund et al.	1993	Sweden	Caucasian	PB	86	99	35	38	13	33	54	12	Yes
Vartiainen et al.	2000	Finland	Caucasian	PB	167	172	64	81	22	73	76	23	Yes
Senti et al.	2001	Spain	Caucasian	PB	312	317	134	139	39	141	137	39	Yes
Yusuf et al.	2004	Several	Asian	PB	1092	1187	351	498	243	389	531	267	Yes
Low et al.	2005	USA	Caucasian	HB	204	260	89	86	29	114	111	35	Yes
Helgadottir et al.^1^	2007	USA	Caucasian	PB	875	447	370	399	106	174	221	52	Yes
Helgadottir et al.^2^	2007	USA	Caucasian	PB	933	468	359	441	133	194	213	61	Yes
Samani et al.^1^	2007	Germany	Caucasian	PB	1126	1277	447	529	150	522	593	162	Yes
Samani et al.^2^	2007	Germany	Caucasian	PB	722	1643	293	328	101	662	753	228	Yes
Meng et al.	2008	Ireland	Caucasian	FB	482	622	203	226	53	261	292	69	Yes
Meiner et al.	2008	USA	Caucasian	PB	505	559	187	228	90	216	260	83	Yes
Serre et al.	2008	Several	Mixed	PB	789	859	335	337	117	354	402	103	Yes
Morgan et al.	2008	USA	Caucasian	HB	807	637	322	377	108	256	304	77	Yes
Assimes et al.	2008	USA	Caucasian	PB	505	514	162	187	83	144	183	130	Yes
Vennemann et al.	2008	Germany	Caucasian	PB	793	1121	311	379	103	430	528	163	Yes
Sutton et al.	2008	USA	Caucasian	FB	1575	970	545	570	183	297	347	86	Yes
Martinelli et al.	2008	Italy	Caucasian	PB	1106	383	437	501	168	145	191	47	Yes
Iakoubova et al.	2008	Scottland	Caucasian	PB	481	1080	104	137	35	256	204	59	Yes
Stewart et al.	2009	Canada	Caucasian	HB	1540	1455	183	695	662	205	616	634	Yes
Luke et al.	2009	Austria	Caucasian	HB	505	782	73	254	178	102	373	307	Yes
Bare et al.	2010	Costa Rican	Caucasian	PB	1987	2147	785	952	250	896	966	285	Yes
Assimes et al.^1^	2010	U.S.A	Mixed	PB	505	514	192	220	93	161	213	140	Yes
Assimes et al.^2^	2010	Germany	Caucasian	HB	793	1121	311	379	103	430	528	163	Yes
Assimes et al.^3^	2010	U.S.A	Mixed	HB	1575	970	561	670	344	306	433	231	Yes
Assimes et al.^4^	2010	Iceland	Caucasian	PB	4313	24,952	2131	1779	403	11,813	10,689	2450	Yes
Assimes et al.^5^	2010	Finland	Caucasian	PB	167	172	64	81	22	73	76	23	Yes
Assimes et al.^6^	2010	U.S.A	Mixed	FB	378	2652	108	182	88	679	1105	868	Yes
Assimes et al.^7^	2010	Germany	Caucasian	PB	722	1643	293	328	101	662	753	228	Yes
Assimes et al.^8^	2010	Germany	Caucasian	HB	1126	1277	447	529	150	522	593	162	Yes
Assimes et al.^9^	2010	U.S.A	Caucasian	CB	505	559	187	228	90	216	260	83	Yes
Assimes et al.^10^	2010	Mixed	Caucasian	H-CB	789	859	335	337	117	354	402	103	Yes
Assimes et al.^11^	2010	Mixed	Asian	H-CB	1092	1187	351	498	243	389	531	267	Yes
Assimes et al.^12^	2010	Ireland	Caucasian	FB	482	622	203	226	53	261	292	69	Yes
Assimes et al.^13^	2010	Sweden	Caucasian	PB	86	99	35	38	13	33	54	12	Yes
Assimes et al.^14^	2010	U.S.A	Caucasian	HB	875	447	370	399	103	174	221	52	Yes
Assimes et al.^15^	2010	U.S.A	Caucasian	HB	204	260	89	86	29	114	111	35	Yes
Assimes et al.^16^	2010	U.S.A	Caucasian	HB	807	637	322	377	108	256	304	77	Yes
Assimes et al.^17^	2010	U.S.A	Caucasian	HB	933	468	359	441	133	194	213	61	Yes
Assimes et al.^18^	2010	Spain	Caucasian	CB	312	317	134	139	39	141	137	39	Yes
Assimes et al.^19^	2010	Italy	Caucasian	HB	1106	383	437	501	168	145	191	47	Yes
Assimes et al.^20^	2010	U.K.	Caucasian	CB	1922	2933	792	890	240	1242	1299	392	Yes
Bhanushali et al.	2011	India	Asian	HB	227	150	70	111	46	33	80	37	Yes
Peng et al.	2012	China	Asian	HB	289	522	69	149	71	139	262	121	Yes
Wu et al.^1^	2012	China	Asian	HB	356	568	104	164	88	168	268	132	Yes
Wu et al.^2^	2012	China	Asian	HB	114	568	16	68	30	168	268	132	Yes
Wu et al.	2014	China	Asian	HB	288	346	74	141	73	101	166	79	Yes
Hamidizadeh et al.	2015	Iran	Caucasian	HB	100	100	35	48	17	63	27	10	No
Vishnuprabu et al.	2015	India	Asian	HB	510	532	107	252	151	121	251	160	Yes
Hubacek et al.	2016	Czech	Caucasian	HB	1889	1191	691	856	302	440	543	195	Yes
Vatte et al.	2016	Saudi Arabia	Asian	HB	1002	984	277	513	212	286	464	234	Yes

1–20: represents different studies in one publication; *HB* hospital based study, *PB* population based study, *FB* family based study, *CB* community based study, *H-CB* hospital and community based study, *HWE* Hardy-Weinberg equilibrium. Mix: the original studies didn’t clarify the race of the subjects or mixed races

### Quantitative synthesis

**Fig. 2:**
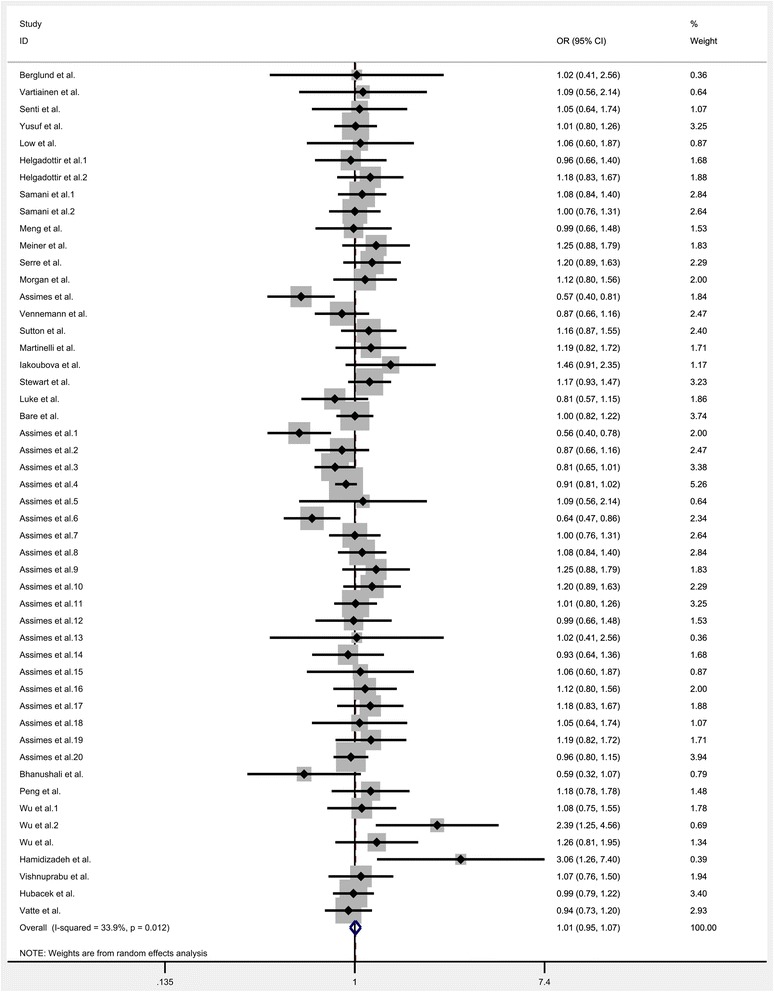
Forest plot of the association between KIF6 rs20455 gene polymorphism and CHD risk (under homozygote model)

**Fig. 3:**
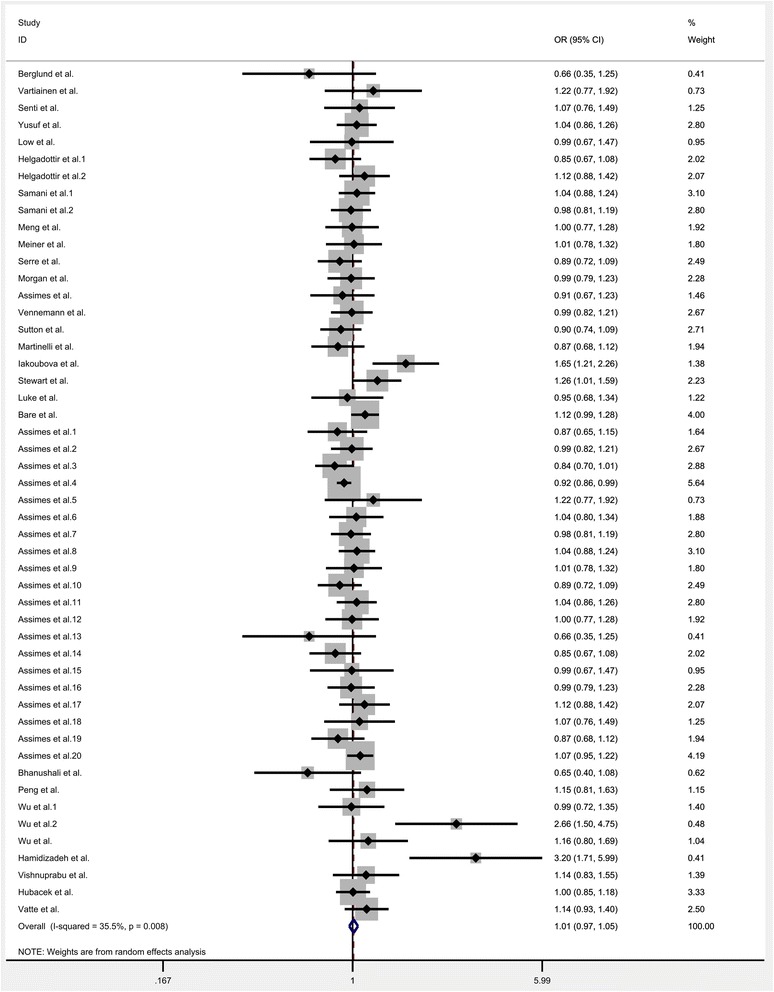
Forest plot of the association between KIF6 rs20455 gene polymorphism and CHD risk (under heterozygote model)

**Fig. 4:**
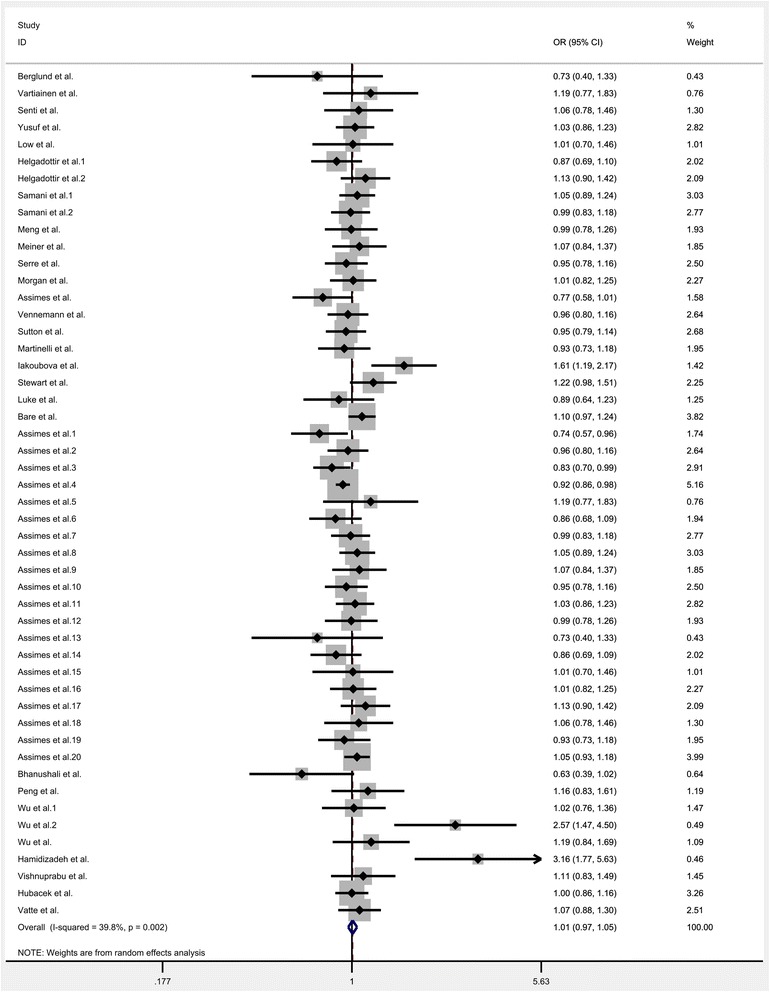
Forest plot of the association between KIF6 rs20455 gene polymorphism and CHD risk (under dominant model)

**Fig. 5:**
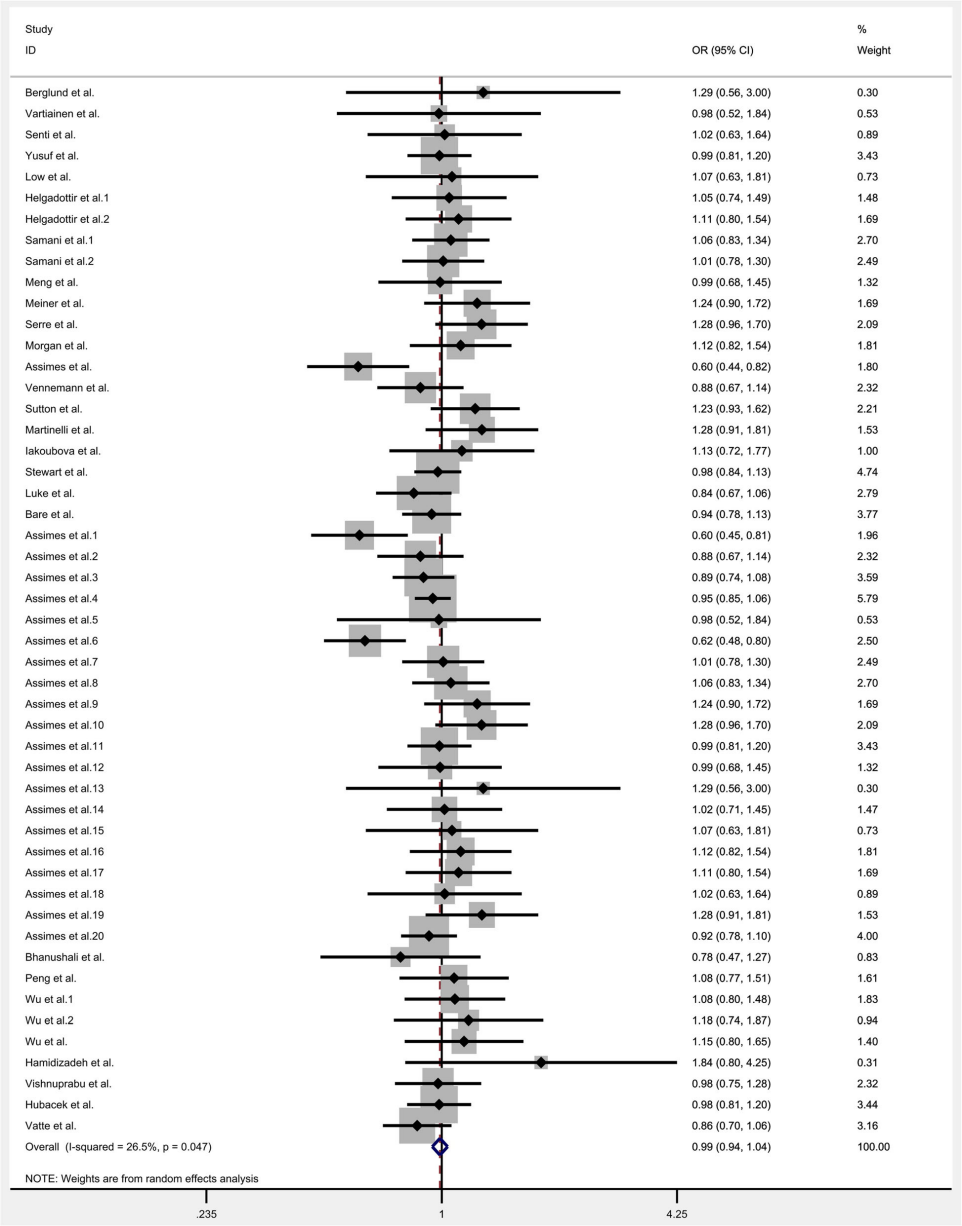
Forest plot of the association between KIF6 rs20455 gene polymorphism and CHD risk (under recessive model)

**Fig. 6:**
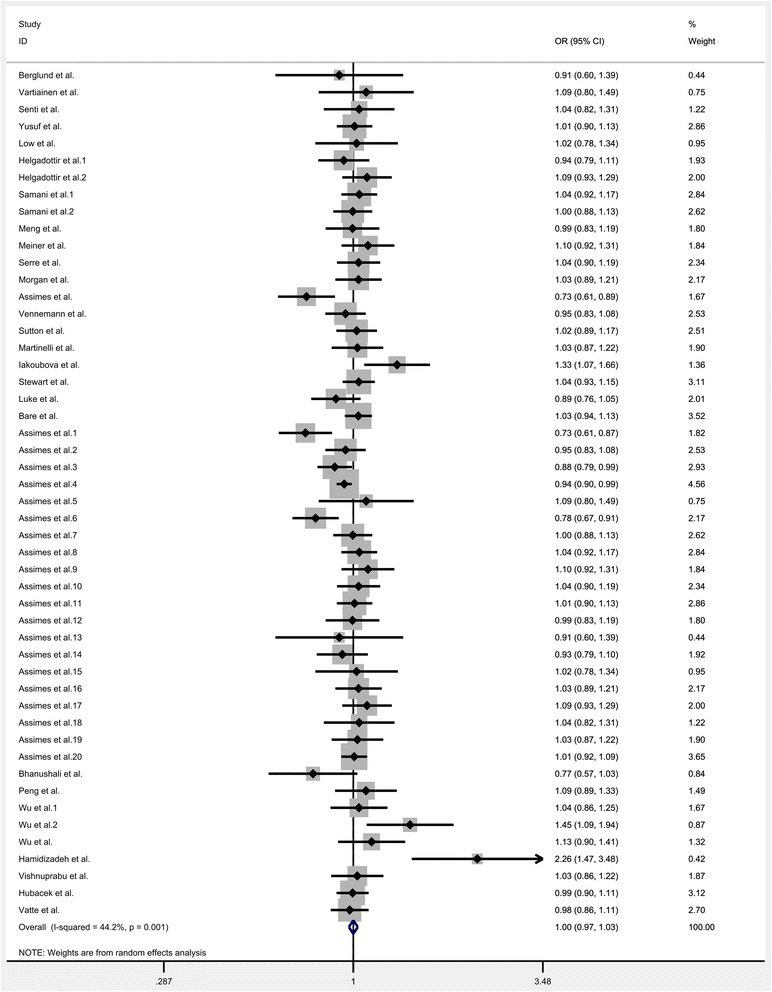
Forest plot of the association between KIF6 rs20455 gene polymorphism and CHD risk (under allele comparison model)

All the eligible data were calculated and significant heterogeneity was detected under homozygote (*I*^*2*^ = 33.9%; P_heterogeneity_ = 0.012), heterozygote (*I*^*2*^ = 35.5%; P_heterogeneity_ = 0.008), dominant (*I*^*2*^ = 39.8; P_heterogeneity_ = 0.002), recessive (*I*^*2*^ = 26.5%; P_heterogeneity_ = 0.047) and allele comparison model (*I*^*2*^ = 44.2%; P_heterogeneity_ = 0.001) between this gene variation and the risk of CHD. So, random-effect model was used to calculate the statistical parameters. Overall, there were no significant association existed between KIF6 rs20455 polymorphism and the risk of CHD (Homozygote model: OR = 1.007, 95% CI =0.952–1.066, *P* = 0.801, Fig. 2; Heterozygote model: OR = 1.009, 95% CI = 0.968–1.052, *P* = 0.636, Fig. 3; Dominant model: OR = 1.007, 95% CI = 0.966–1.048, *P* = 0.753, Fig. 4; Recessive model: OR = 0.989, 95% CI = 0.943–1.037, *P* = 0.655, Fig. 5; Allele comparison model: OR = 1.00, 95% CI = 0.971–1.030, *P* = 0.988, Fig. 6). Furthermore, we explored the subgroup analyses by ethnicity and source of control. All the results were listed in Table 2.

**Table 2:** Main results of pooled ORs with 95% CI in the meta-analysis

Variables	No.	P_heterogneity_	Analysis model	OR (95% CI)	P	P_Begg’s_	P_Egger’s_
Homozygote model							
*Total*	50	0.012	Random model	1.007 (0.952–1.066)	0.801	0.106	0.108
*Ethnicity*							
Caucasian	37	0.45	Fixed model	1.012 (0.964–1.063)	0.622		
Asian	9	0.158	Fixed model	1.038 (0.933–1.154)	0.494		
Mixed	4	0.004	Random model	0.771 (0.57–1.043)	0.0731		
*Source of control*							
PB	20	0.038	Random model	0.981 (0.895–1.076)	0.691		
HB	21	0.096	Fixed model	1.027 (0.956–1.103)	0.891		
FB	4	0.038	Fixed model	0.907 (0.767–1.072)	0.016		
CB	3	0.427	Fixed model	1.019 (0.872–1.189)	0.816		
H-CB	2	0.368	Fixed model	1.073 (0.895–1.286)	0.446		
Heterozygote model							
*Total*	50	0.008	Random model	1.009 (0.968–1.052)	0.636	0.089	0.070
*Ethnicity*							
Caucasian	37	0.035	Random model	0.955 (0.963–1.029)	0.790		
Asian	9	0.071	Fixed model	1.089 (0.995–1.191)	0.065		
Mixed	4	0.639	Fixed model	0.893 (0.799–0.999)	0.047		
*Source of control*							
PB	20	0.067	Random model	0.979 (0.938–1.021)	0.316		
HB	21	0.004	Random model	1.040 (0.956–1.132)	0.356		
FB	4	0.807	Fixed model	0.966 (0.859–1.085)	0.558		
CB	3	0.924	Fixed model	1.064 (0.957–1.183)	0.254		
H-CB	2	0.265	Fixed model	0.967 (0.841–1.112)	0.637		
Dominant model							
*Total*	50	0.002	Random model	1.007 (0.966–1.048)	0.753	0.061	0.058
*Ethnicity*							
Caucasian	37	0.034	Random model	1.013 (0.970–1.057)	0.568		
Asian	9	0.054	Fixed model	1.071 (0.984–1.165)	0.112		
Mixed	4	0.508	Fixed model	0.854 (0.770–0.947)	0.003		
*Source of control*							
PB	20	0.026	Random model	0.991 (0.932–1.055)	0.786		
HB	21	0.002	Random model	1.040 (0.958–1.129)	0.346		
FB	4	0.820	Fixed model	0.948 (0.848–1.059)	0.342		
CB	3	0.986	Fixed model	1.053 (0.953–1.164)	0.310		
H-CB	2	0.551	Fixed model	0.993 (0.871–1.132)	0.917		
Recessive model							
*Total*	50	0.047	Random model	0.989 (0.943–1.037)	0.655	0.025	0.040
*Ethnicity*							
Caucasian	37	0.541	Fixed model	1.002 (0.959–1.048)	0.919		
Asian	9	0.819	Fixed model	0.983 (0.898–1.075)	0.705		
Mixed	4	<0.001	Random model	0.811 (0.592–1.111)	0.191		
*Source of control*							
PB	20	0.040	Random model	0.982 (0.902–1.069)	0.668		
HB	21	0.796	Fixed model	0.989 (0.919–1.064)	0.715		
FB	4	0.004	Random model	0.924 (0.661–1.291)	0.643		
CB	3	0.287	Fixed model	1.009 (0.843–1.209)	0.883		
H-CB	2	0.142	Fixed model	1.099 (0.856–1.412)	0.395		
Allele comparison model							
*Total*	50	0.001	Random model	1.00 (0.971–1.030)	0.988	0.052	0.066
*Ethnicity*							
Caucasian	37	0.067	Fixed model	0.999 (0.977–1.022)	0.950		
Asian	9	0.186	Fixed model	1.022 (0.968–1.079)	0.428		
Mixed	4	0.009	Random model	0.855 (0.742–0.985)	<0.001		
*Source of control*							
PB	20	0.004	Random model	0.990 (0.943–1.040)	0.690		
HB	21	0.017	Random model	1.015 (0.967–1.066)	0.547		
FB	4	0.045	Random model	0.877 (0.691–1.113)	0.361		
CB	3	0.653	Fixed model	1.025 (0.953–1.102)	0.507		
H-CB	2	0.776	Fixed model	1.019 (0.931–1.115)	0.687		

*No*. number of studies, *OR* odds ratio, *95% CI* 95% confidence interval, *HB* hospital based study, *PB* population based study, *FB* family based study, *CB* community based study, *H-CB* hospital and community based study

### Sensitivity analysis

The sensitivity analysis was performed to evaluate the influence of each individual study on the pooled OR by omitting every single study. The analysis results reflected that our results were statistically stable and reliable.

### Publication bias

**Fig. 7:**
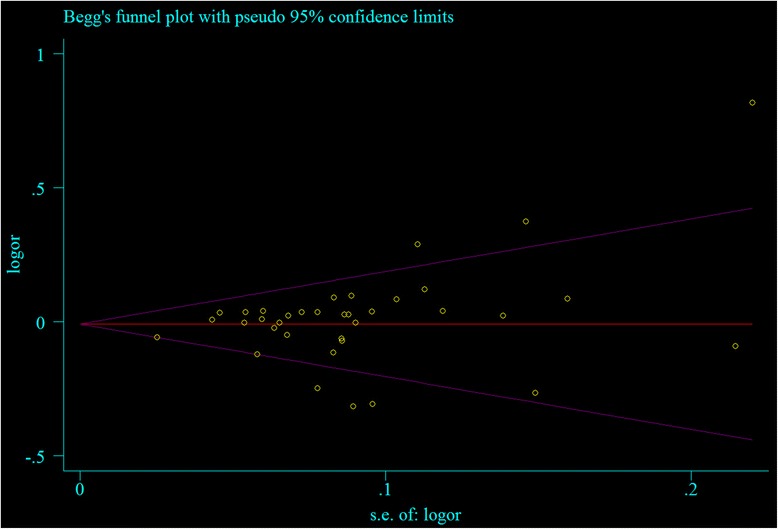
Begg’s test of the association between KIF6 rs20455 gene polymorphism and CHD risk (under allele comparison model)

**Fig. 8:**
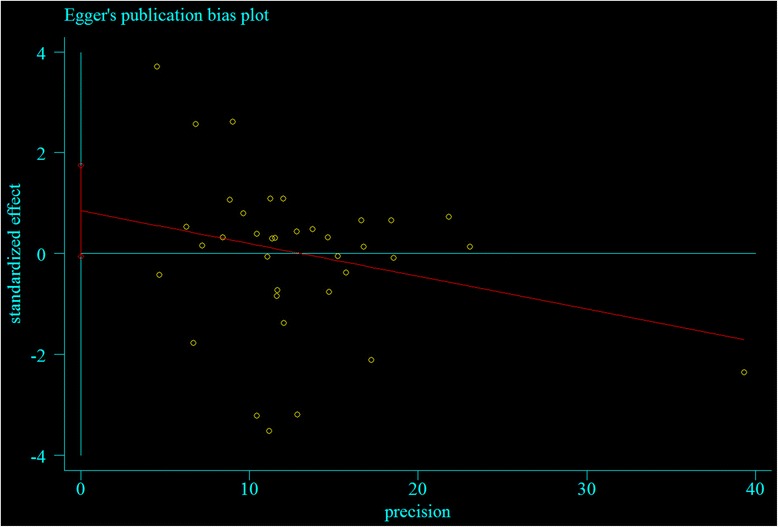
Egger’s test the association between KIF6 rs20455 gene polymorphism and CHD risk (under allele comparison model)

There was no significant publication bias found in the meta-analysis, reflected by *P* values from Begg’s correlation (Heterozygote model: *P* = 0.089; Dominant model: *P* = 0.061; Allele comparison model: *P* = 0.052, Fig. 7) and Egger’s regression (Heterozygote model: *P* = 0.070; Dominant model: *P* = 0.058; Allele comparison model: *P* = 0.066, Fig. 8). However, significant publication bias found in the meta-analysis, reflected by P values from Begg’s correlation (Homozygote model: *P* = 0.046; Recessive model: *P* = 0.025) and Egger’s regression (Homozygote model: *P* = 0.041; Recessive model: *P* = 0.040). All the results are listed in Table 2.

## Discussion

Large sample and unbiased epidemiological studies of predisposition genes polymorphisms could provide insight into the in vivo relationship between candidate genes and complex diseases. Many epidemiological studies have investigated the relationship between the KIF6 rs20455 polymorphism and the risk of CHD, but because of small sample size and the low statistical power of individual studies, results have been contradictory. In this present study, we searched all eligible studies to date and got the precise result if KIF6 rs20455 polymorphism could contribute to the risk of CHD. To the best of our knowledge, our present work was the most comprehensive one through enrolling all eligible studies.

Herein, we included 50 individual studies, including 40,059 cases and 64,032 controls. Overall, there was no association between KIF6 rs20455 polymorphism and CHD risk. Hamidizadeh et al. found that significant association was found between this gene polymorphism and CHD risk among Caucasian populations [43], and the result was verified in another study through enrolling 143,000 subjects [40]. However, no association was found in a meta-analysis, among South Asians, African-Americans, Hispanics, East Asians, and mixed decedent populations [39]. Furthermore, other recent studies were also found no association existed between this gene polymorphism and CHD risk [25, 26, 47–49]. When we got the subgroup analyses by ethnicity, there was also no association found among Caucasian and Asian populations. While decreased risk of this gene polymorphism and CHD risk was found among mixed populations. Of note, mixed populations means the original studies didn’t clarify the race of the subjects or mixed races. This result may be not provided some useful information for clinical deeds. So, further studies should be performed with clearly race or ethnicity stated in their work.

Publication bias was found in some genetic models. The explanations might arise from some aspects. First, our meta-analysis took into consideration only fully published data, and the abstract and conference papers were excluded. Second, this meta-analysis only focused on papers published in Chinese and English language, and some eligible studies which were reported in other languages might be missed. Third, positive results tend to be accepted by journals while negative results are often rejected or not even submitted. We should point out that the publication bias might partly account for the results, but which were not affected deeply. When we adjusted the results using the trim and fill method, the adjusted risk estimate was attenuated but remained significant, indicating the stability of our results.

Some limitations of this meta-analysis should be addressed. Firstly, heterogeneity is a potential problem when interpreting all the results of meta-analysis. Although we minimized the likelihood by performing a careful search for published studies, using the explicit criteria for study inclusion, the significant between-study heterogeneity still existed in most of comparison. The presence of heterogeneity can result from differences in the age distribution, selection of controls, prevalence lifestyle factors and so on. Secondly, only published studies were included in this meta-analysis. Therefore, potential publication bias was existed in some genetic models. Despite the limitations, our meta-analysis significantly increased the statistical power based on substantial data from different studies. The sensitivity analyses outcomes reflected that our results were statistically stable and reliable.

In conclusion, this present meta-analysis suggests that carriers of KIF6 rs20455 polymorphism may irrelative to the risk of CHD. We also observed no compelling evidence of an association between the KIF6 rs20455 SNP and CHD in multiple race/ethnic groups. These findings do not support the clinical utility of testing for the KIF6 rs20455 polymorphism in the primary prevention of CHD and indirectly question whether genotype information at this locus is able to identify subjects most likely to benefit from the use of statins.

## Abbreviations

CHD: Coronary heart disease
CHS: Cardiovascular Health Study
CI: Confidence interval
KIF6: Kinesin-like protein 6
OR: Odds ratio
PROSPER: Prospective Study of Pravastatin in the Elderly at Risk
SNPs: Single nucleotide polymorphisms
WHS: the Women’s Health Study
